# Successful treatment with selpercatinib after pralsetinib-related pneumonitis and intracranial failure in a patient with RET-rearranged nonsmall cell lung cancer

**DOI:** 10.1097/CAD.0000000000001590

**Published:** 2024-03-08

**Authors:** Valeria Cognigni, Giulia Claire Giudice, Francesca Bozzetti, Gianluca Milanese, Ilaria Moschini, Miriam Casali, Giulia Mazzaschi, Marcello Tiseo

**Affiliations:** a Department of Medical Oncology, Università Politecnica delle Marche, Ancona; b Department of Medicine and Surgery, University of Parma; c Medical Oncology Unit; d Neuroradiology Unit; e Radiology Unit, University Hospital of Parma, Parma; f Radiotherapy Unit, AUSL Piacenza, Piacenza; g Medical Oncology Unit, Azienda Socio-Sanitaria Territoriale di Lodi, Lodi, Italy

**Keywords:** intracranial efficacy, nonsmall cell lung cancer, pulmonary toxicity, RET-rearrangement, selpercatinib

## Abstract

Pralsetinib and selpercatinib are two highly potent and selective rearranged during transfection (RET) inhibitors that substantially improved the clinical outcome of patients with RET-rearranged non-small cell lung cancer. Treatment with one RET inhibitor after failure of the other is generally not recommended because of cross-resistance mechanisms. We report the case of a patient affected by metastatic RET-rearranged non-small cell lung cancer who experienced long-lasting disease control with pralsetinib. After 13 months from treatment start, the patient developed recurrent drug-related pneumonitis, requiring temporary interruptions and dose reductions and eventually failing to control the disease. Selpercatinib was then started as an off-label treatment, allowing both clinical and radiological intracranial disease control. Selpercatinib was well-tolerated at full dosage, and no pulmonary event occurred. In our case report, after pralsetinib dose reduction due to pulmonary toxicity, the therapeutic switch to selpercatinib allowed the patient to receive a full-dose treatment, eventually restoring disease control. Our case report and a few literature data suggest that switching from pralsetinib to selpercatinib may represent a therapeutic opportunity, especially for patients with brain metastases.

## Introduction

In the last 10 years, targeted therapy has dramatically changed the history and prognosis of advanced oncogene-addicted non-small cell lung cancer (NSCLC). The incidence of rearranged during transfection (*RET*) proto-oncogene rearrangements in NSCLC is about 1–2% [1]. The chromosomal rearrangements between *RET* gene and another gene partner, such as kinesin family 5B and coiled-coil domain containing 6, lead to a constitutive oncogenic activation, that activates downstream signaling of mitogen-activated protein kinase (MAPK), janus kinase-signal transducer and activator of transcription and phosphatidylinositol 3-kinase-protein kinase B-mammalian target of rapamycin molecular pathways and promotes cancer cell proliferation and survival [2].

Patients with *RET*-rearranged NSCLC are usually young, light or never smokers, with adenocarcinoma histology and a high incidence of pleural involvement and brain metastases [2]. Two highly potent and selective RET inhibitors have been recently approved and significantly improved NSCLC patients’ clinical outcomes. Indeed, pralsetinib and selpercatinib demonstrated a robust efficacy and manageable safety profile in the phase 1/2 ARROW (NCT03037385) and LIBRETTO-001 (NCT03157128) studies [3,4]. Recently, in the phase 3 LIBRETTO-431 (NCT04194944) trial selpercatinib led to significantly longer progression-free survival than platinum-based chemotherapy with or without pembrolizumab in the first-line treatment of advanced *RET*-rearranged NSCLC [5].

Pralsetinib and selpercatinib are not recommended after failure of another RET tyrosine kinase inhibitor (TKI), because of superimposable resistance mechanisms [6,7]. Here, we report a case of a *RET*-rearranged metastatic NSCLC patient, successfully treated with selpercatinib after disease progression to pralsetinib.

## Case description

In December 2018, a 58-year-old never-smoker woman without significant pathological history, presented to the emergency department with dizziness and occipital headache. A brain magnetic resonance imaging(MRI) was performed describing three peculiar cystic-like brain lesions in the parietal lobe of each side. As previously described by Facchinetti *et al*. [8], she underwent brain resection of the major lesion on the left occipital lobe, with the diagnosis of metastatic brain lesion from lung adenocarcinoma, with negative programmed cell death ligand 1 expression and the presence of a rearrangement of RET exon 12—kinesin family 5B exon 15. A whole-body computed tomographic (CT) scan documented a mass in the upper left lung lobe with bilateral nodal involvement, with clinical staging determined as cT2aN3M1c, according to tumor-node-metastasis staging VIII edition.

Based on the stage of the disease, the molecular characteristics and the good clinical condition, a first-line chemotherapy treatment with cisplatin-pemetrexed was started; in addition, stereotaxic radiotherapy on the brain and left lung lesions was performed, obtaining a tumor response. In October 2019, both brain MRI and 18F-fluorodeoxyglucose positron emission tomography-CT scans showed disease progression. She was then enrolled in a clinical trial and received cabozantinib for 3 months, with an initial intracranial and lung disease response but subsequent progression. Consequently, therapy with pralsetinib as compassionate use, at a dose of 400 mg per day, was started in January 2020 and continued for 3 years, allowing major and prolonged disease control in all disease sites. After 13 months from treatment start, the patient developed a drug-related grade 2 (Common Terminology Criteria for Adverse Events [CTCAE] v5.0) pneumonitis that required steroid therapy, temporary interruption and dose reductions of up to 200 mg per day. To control the disease on sites of oligo-progression, additional stereotactic radiotherapy was performed on brain and lung lesions.

**Fig. 1:**
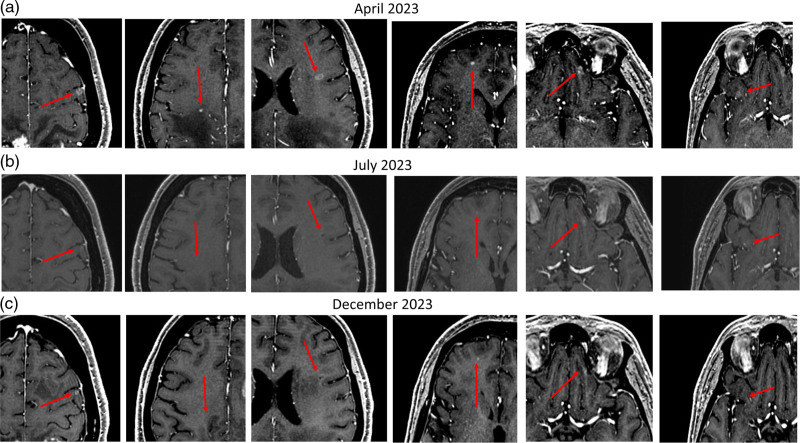
Brain MRI, axial sections of T1-weighted images after gadolinium administration. (a) during treatment with pralsetinib at a reduced dose, progression of the disease was documented by the appearance of multiple brain metastases (red arrows) in different cerebral lobes characterized by both cortical-subcortical and deep distribution, small size without significant vasogenic edema. (b) three months after starting selpercatinib, partial response was proved by the reduction of the size of the multiple cerebral metastases; some lesions disappeared such as the lesion of the deep white matter of the right frontal lobe and the cortical-subcortical lesion of the left inferior frontal gyrus. (c) six months after starting selpercatinib, the persistent cerebral response was maintained for all the cerebral lesions.

In May 2023, radiological progression disease was detected, with an increase of the left lower lung lesion and the appearance of multiple brain metastases (Fig. 1a). Because of the number of brain lesions and the previous radiotherapy treatments, a stereotactic strategy was excluded. Considering the patient’s good performance status, the mainly cerebral disease burden and the poor activity of chemotherapy on brain lesions, selpercatinib was started as an off-label treatment. The brain MRI performed after 3 months since selpercatinib start showed a brain tumor response (Fig. 1b), while the CT scan showed stable lung disease. Selpercatinib was well-tolerated, only burdened by xerostomia; no other pulmonary event was detected. The treatment is still ongoing after 8 months with maintenance of clinical and radiological disease control (Fig. 1c).

## Discussion

We described an interesting case regarding both the intracranial and extracranial efficacy of selpercatinib after the failure of pralsetinib in a patient affected by *RET*-rearranged metastatic NSCLC.

Pralsetinib and selpercatinib are RET inhibitors that have been approved based on the results of two phase 1/2 studies: they showed a remarkable intracranial activity and significantly improved clinical outcome of *RET*-rearranged advanced NSCLC patients, both after the failure of a platinum-based treatment and as first-line treatment [3–5]. Increasing evidence is bringing out the need to anticipate TKI treatment in first-line over chemotherapy [5] but, in the literature, data about TKI after TKI failure or comparison between two TKIs for *RET*-rearranged NSCLC are still lacking.

A TKI treatment after failure to another TKI is generally not recommended because of cross-resistance mechanisms [6,7]. Acquired resistance to RET-TKI can develop through on-target molecular mechanisms or *RET*-independent alterations, that may concern mesenchymal-epithelial transition factor amplifications, Kirsten rat sarcoma viral oncogene homolog or other mitogen-activated serine/threonine protein kinase (*MAPK*) genes activating mutations [2,9].

After 13 months from pralsetinib start, our patient experienced pulmonary toxicity that required steroid administration, drug interruption and subsequent dose reduction. The incidence of pralsetinib-related pneumonitis of any grade is about 10–12% and is the most common adverse event leading to permanent treatment discontinuation [3,10]; a complete resolution of toxicity is possible in 64% of patients with steroid administration. Differently, pneumonitis is rare in patients receiving selpercatinib compared to pralsetinib (about 4% of patients) [4].

The therapeutic alternative for our patient, after the TKI failure, would have been chemotherapy and whole-brain radiotherapy, considering the large number of brain metastatic lesions. Although pralsetinib allowed a long-lasting disease control of 40 months, because the patient was not receiving a full dosage treatment with pralsetinib and had a prevalent brain progression, we hypothesized that the disease resistance was not strictly related to a biological mechanism, but to an inadequate pharmacokinetic. Considering these aspects, a re-treatment with a RET-TKI was proposed, obtaining a radiological benefit. Switch to selpercatinib allowed us to administer a drug at the right dose without pulmonary toxicity and achieving an intracranial benefit.

To the best of our knowledge, only two similar cases were reported in the literature. Tsui *et al*. [11] described the clinical and radiological benefits of switching pralsetinib to selpercatinib in a patient with a progressive leptomeningeal disease. At the time of disease progression to pralsetinib, the patient also received stereotactic radiosurgery on eight subcentimetric brain metastases and oral steroids to manage neurological symptoms; the authors suggested that selpercatinib is the main responsible for intracranial response, given the improvement of leptomeningeal disease and the subsequent steroid discontinuation. D’Arienzo *et al*. [12] illustrated a case of complete intracranial response to selpercatinib after the development of leptomeningeal and brain metastases and pralsetinib discontinuation due to drug-related pneumonitis. The authors, similarly to the present case, highlighted the intracranial activity of selpercatinib without drug-related pneumonitis and dose reduction.

### Conclusion

Based on our own experience, in accordance with two other case reports in the literature, subsequent treatment with RET-TKI after one TKI may represent a potential therapeutic possibility for those patients affected by *RET*-rearranged NSCLC brain metastases and who received the first TKI at a reduced dosage due to adverse events.

## Acknowledgements

The patient provided consent to this publication. Written informed consent was obtained from the patient for publication of this case report and accompanying images.

Conception and design: M.T. Provision of study materials or patients: G.C.G., G.M., F.B., G.M., I.M. and M.C. Collection and assembly of data: V.C., G.C.G. and M.T. Manuscript writing: All authors. Final approval of manuscript: All authors.

### Conflicts of interest

M.T. received speakers’ and consultants’ fee from Astra-Zeneca, Pfizer, Eli-Lilly, BMS, Novartis, Roche, MSD, Boehringer Ingelheim, Otsuka, Takeda, Pierre Fabre, Amgen, Merck, Sanofi. M.T. received institutional research grants from Astra-Zeneca, Boehringer Ingelheim. For the remaining authors, there are no conflicts of interest.
